# Genetic variations of aldehyde dehydrogenase 2 and alcohol dehydrogenase 1B are associated with the etiology of atrial fibrillation in Japanese

**DOI:** 10.1186/s12929-016-0304-x

**Published:** 2016-12-07

**Authors:** Yukiko Nakano, Hidenori Ochi, Yuko Onohara, Akinori Sairaku, Takehito Tokuyama, Hiroya Matsumura, Shunsuke Tomomori, Michitaka Amioka, Naoya Hironomobe, Chikaaki Motoda, Nozomu Oda, Kazuaki Chayama, Che-Hong Chen, Eric R. Gross, Daria Mochly-Rosen, Yasuki Kihara

**Affiliations:** 1 Department of Cardiovascular Medicine, Hiroshima University Graduate School of Biomedical & Health Sciences, 1-2-3 Kasumi, Minami-ku, Hiroshima 734-8551 Japan; 2 Laboratory for Digestive Diseases, Center for Integrative Medical Sciences, RIKEN, Hiroshima, Japan; 3 Department of Gastroenterology and Metabolism, Applied Life Science, Institute of Biomedical & Health Science, Hiroshima University , Hiroshima, Japan; 4 Department of Chemical & Systems Biology, School of Medicine, Stanford University, Stanford, CA USA; 5 Department of Anesthesiology, Perioperative and Pain Management, School of Medicine, Stanford University, Stanford, CA USA

**Keywords:** Atrial fibrillation, Alcohol, *ALDH2*, *ADH1B*, Single nucleotide polymorphism

## Abstract

**Background:**

Alcohol consumption and oxidative stress are well-known risk factors for developing atrial fibrillation (AF). Single nucleotide polymorphisms (SNPs) of alcohol dehydrogenase (*ADH1B*) and aldehyde dehydrogenase 2 (*ALDH2*) genes encoding enzymes of alcohol and reactive aldehyde metabolism, respectively, are prevalent among East Asians. Here, we examined whether these SNPs were associated with AF in Japanese patients.

**Methods and results:**

Five hundred seventy-seven Japanese patients with AF undergoing catheter ablation and 1935 controls at Hiroshima University Hospital were studied. Alcohol consumption habits, medical history, electrocardiogram (EKG), electrophysiology and cardiac echocardiography were reviewed. Patients were also genotyped for *ALDH2* (rs671) and *ADH1B* (rs1229984). A significant linear correlation was found between *ALDH2* genotype and mean alcohol intake (*P* = 1.7 × 10^−6^). Further, *ALDH2* (rs671) was associated with AF (*P* = 7.6 × 10^−4^, odds ratio [OR] = 0.6). Frequency of the ALDH2 SNP allele A which limits acetaldehyde metabolism was lower in patients with AF (18.8%) than in controls (23.5%). In contrast, we found that the frequencies of the *ADH1B* SNP genotypes were similar in patients with AF and in controls. Subset analysis among the 182 patients with lone AF and 914 controls (control II) (<60 years of age and without hypertension), both *ALDH2* and *ADH1B* SNPs were significantly associated with AF (*P* = 0.013, OR = 0.7; *P* = 0.0007, OR = 1.4, respectively). The frequency of the dysfunctional allele A of *ALDH2* was significantly lower and the dysfunctional allele G of *ADH1B* was significantly higher in patients with lone AF than in control II (*ALDH2* A allele frequency = 0.176 vs 0.235, OR = 1.3, *P* = 0.013, *ADH1B* SNP G allele frequency = 0.286 vs 0.220, OR = 1.4, *P* = 0.0007).

**Conclusions:**

When considering all patients enrolled, the dysfunctional *ALDH2* allele was negatively associated with AF. When examining a subset of patients with lone AF, the dysfunctional *ALDH2* allele was negatively associated with AF and the slower metabolizing *ADH1B* allele was positively associated with AF. Hence*,* prolonged metabolic conversion of alcohol to acetaldehyde may be associated with the occurrence of AF in the Japanese and other East Asian populations.

## Background

Atrial fibrillation (AF) is the most common arrhythmia, with many risk factors being reported, including aging, male sex, hypertension, valvular diseases, left ventricular dysfunction, obesity, sleep apnea, and alcohol consumption [1]. High alcohol consumption is associated with the occurrence of AF. Some meta-analyses have identified a dose–response relationship between alcohol consumption and AF risk, and other recent studies have reported that even moderate alcohol consumption is a risk factor for AF [2, 3].

Two enzymes are mainly involved in alcohol metabolism. Alcohol is first metabolized to acetaldehyde by alcohol dehydrogenase 1B (*ADH1B*), and then to acetic acid by aldehyde dehydrogenase 2 (*ALDH2*) [4]. *ALDH2* is an enzyme not only for alcohol metabolism but also for catalyzing the oxidation of an aldehyde that is a lipid peroxidation product, such as 4-hydroxy-2-nonenal (4-HNE), and other aldehydes. Thus, ALDH2 besides alcohol metabolism, reduces the damage of reactive oxygen species (ROS) and protects against oxidative stress [5, 6]. Amino acid coding single nucleotide polymorphisms (SNPs) of *ADH1B* (G/A, rs1229984) and *ALDH2* (G/A, rs671) are widely known, and the *ADH1B* G and *ALDH2 A* alleles of these SNPs have notably decreased enzymatic activities. The dysfunctional G allele of *ADH1B* results in slower conversion of alcohol to acetaldehyde and the dysfunctional A allele of *ALDH2* SNP is associated with deficiency in the conversion of acetaldehyde to acetic acid, hence the accumulation of toxic acetaldehyde because of their low metabolic activities [7, 8]. The *ALDH2* deficiency has been known to be the underlying cause of “Alcohol Flushing Syndrome” [9]. The *ADH1B* and *ALDH2* SNPs are especially common in East Asians [10]. People with the dysfunctional A allele of the *ALDH2* SNP are at risk for many types of systemic disease because of its reduced capacity on both acetaldehyde metabolism and protection against oxidative stress [11]. Importantly, excess amounts of ROS has been known to be associated with AF by their effects on ion channels, cell coupling, and molecular mechanisms [12].

In this study, we investigated the association of *ADH1B* and *ALDH2* SNPs with AF in Japanese populations because of their involvement with alcohol metabolism and for metabolizing reactive aldehydes produced during ROS production.

## Methods

### Participants

We enrolled 577 patients with AF (427 males and 130 females, mean age 61 ± 10 years) who were undergoing catheter ablation in Hiroshima University Hospital. We also enrolled 1935 non-AF controls (1563 male, mean age 55 ± 13 years) from Hiroshima University Hospital. The Institutional Ethics Committee of the Graduate School of Biomedical Science at Hiroshima University approved all procedures involving human genome usage. Written informed consent was obtained from all participants.

We genotyped SNPs rs671 of *ALDH2* and rs1229984 of *ADH1B* and compared allele frequencies of these SNPs between AF subjects and non-AF controls. The lone AF was defined as AF diagnosed before the age of 60 years in the absence of hypertension and structural heart disease. We also examined the relationships between genotypes of the 2 SNPs in subgroup of 182 patients with lone AF and 914 controls without hypertension or structural heart disease (Control II).

All subjects underwent polysomnography (Somuno Screen, Fukuda Denshi) on the day before admission, and the apnea hypopnea index was calculated.

We interviewed the 332 out 577 enrolled patients with AF about their daily and weekly alcohol intakes. We converted daily and weekly alcohol intakes into ethanol consumption (g/day) = volume of alcohol intake × (alcohol degree/100) × 0.8 for each patient.

### Genotyping of *ALDH2* (rs671) and *ADH1B* (rs1229984)

Blood samples were obtained from all participants. Genomic DNA was extracted from leukocytes using a QIAamp DNA Blood Mini Kit (QIAGEN, Hilden, Germany) according to the standard protocol. Subsequently, we genotyped SNPs rs671 of *ALDH2* and rs1229984 of *ADH1B* in all participants using the Invader assay, as previously described [13, 14].

For typing the SNP of ALDH2 (rs671), we used the forward primer: GATGTGTTTGGAGCCCAGTC, reverse primer: CCCAACAGACCCCAATCC, Invader oligo: GCGAGTACGGGCTGCAGGCATACACTT, signal prove-G: CGCGCCGAGGgAAGTGAAAACTGTGAGTGTGG, and signal prove-A: ATGACGTGGCAGACaAAGTGAAAACTGTGAGTGTG. For typing the SNP of ADH1B (rs1229984), we used forward primer: CAATTTCAGGAATTTGGGTATG, reverse primer: CACACGTGTTCCCTGAGTGT and Invader oligo: CAGGTTGCCACTAACCACGTGGTCATCTGTGA, signal prove-G: CGCGCCGAGGcGACAGATTCCTACAGCC and signal prove-A: ATGACGTGGCAGACtGACAGATTCCTACAGC.

### Echocardiographic measurements

Transthoracic echocardiographic examinations were performed in all patients with an iE33 ultrasound (Philips Medical Systems, Best, the Netherlands) equipped with a 3.5-MHz transducer at a depth of 16 cm with the patient in the left lateral decubitus position. The left atrial volume index was calculated by dividing the maximal left atrial volume by the body surface area. Left ventricular diameter and wall thickness were measured by two-dimensional echocardiography. Echocardiographic measurements were taken in accordance with the recommendations of the American Society of Echocardiography [15].

### Electrophysiological study

The patients underwent electrophysiological study after pulmonary vein isolation. Three 5-French quadripolar electrode catheters, each with a 5-mm interelectrode distance, were positioned at the high right atrium, His bundle, and right ventricle. Right atria to His (AH) and His to right ventricle (HV) intervals were measured on the baseline electrocardiogram. Sinus node recovery time and atrioventricular node effective refractory period were also determined.

### Statistical analysis

Normally distributed continuous variables are presented as means ± standard deviation. The differences between the three genotypes were analyzed by a linear regression for continuous data. Odds ratios (ORs) and 95% confidence intervals (CIs) are stated as appropriate. To test the genetic association between cases and controls, we used the chi-square test and the Cochran–Armitage trend test. Deviation from the Hardy–Weinberg equilibrium was tested among the cases and controls with an ordinary chi-square test, where a *P*-value of <0.05 was considered to indicate statistical significance.

## Results

**Fig. 1:**
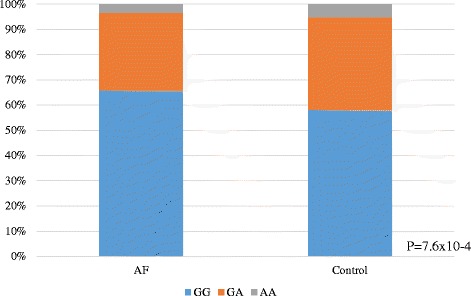
ALDH2 Polymorphism (rs671) in AF Patients and Controls. The frequency of the dysfunctional genotypes with allele A was significantly lower in patients with AF than in controls (*P* = 0.0007)

*ALDH2* (rs671) was significantly negatively associated with AF (*P* = 7.6 × 10^−4^, OR = 1.3). In control group, the GG, GA and AA genotypes were 58.2, 36.7 and 5.1% as compared with 66.0, 30.6 and 3.5% respectively in the AF group (Fig. 1). We found that both the GA and AA genotype of ALDH2 were less common in the AF patient group. The frequency of the dysfunctional allele A was significantly lower in patients with AF than in controls (0.188 vs 0.235 *P* = 0.0007, Table 1). Table 2 shows the relationships between *ALDH2* genotypes and clinical manifestations in patients with AF. We noticed that the frequency of ischemic heart disease was higher in AF patients with AA and GA genotypes than those with GG genotypes. The other clinical characteristics, echocardiographic findings, and electrophysiological study (EPS) findings were all similar for each *ALDH2* genotype.

**Table 1:** ALDH2 Polymorphism (rs671) in AF Patients and Controls

	Genotype distribution			HW-test	allelic model (G vs A)			dominant model (GG + GA vs AA)		recessive model (GG vs GA + AA)	
	GG	GA	AA	*P*	A allele frequency	*P*	OR(95%CI)	*P*	OR(95%CI)	*P*	OR(95%CI)
AF	380	176	20	0.95	0.19	7.6x10^−4^	0.6 (0.4–0.8)	0.1	0.7 (0.4–1.1)	0.8x10^−3^	0.7 (0.6–0.9)
	(66.0%)	(30.6%)	(3.5%)								
Control	1126	710	99	0.34	0.24						
	(58.2%)	(36.7%)	(5.1%)								

The results were tested by the chi-square test and the Cochran–Armitage trend test

The G allele was considered as the reference allele in the allelic model. The A allele was dysfunctional allele

The dysfunctional allele A of ALDH2 SNP rs671 significantly decreased in AF patients (OR 0.6, *P* = 7.0x10-4)

*OR* odds ratio

**Table 2:** Characteristics of AF Patients and ALDH2 Genotype

rs671 (Risk Allele = A)	G/G	G/A	A/A	*P*
No. of patients	380	176	20	
Clinical Characteristics				
Age (year)	62 ± 11	61 ± 10	62 ± 12	0.698
Male gender	275 (72.4%)	138 (78.4%)	14 (70%)	0.377
BMI (kg/m2)	24.1 ± 3.4	24.2 ± 3.3	25.3 ± 3.8	0.380
PAF	232 (61.0%)	91 (51.7%)	11 (55.0%)	0.083
Hypertension	230 (60.6%)	79 (44.9%)	10 (50%)	0.109
Diabetes mellitus	68 (17.8%)	22 (12.5%)	2 (10.0%)	0.266
Ischemic heart disease	7 (1.8%)	12 (6.8%)	2 (10.0%)	0.290
AHI/hour	20.5 ± 13.5	18.9 ± 13.5	17.0 ± 11.4	0.430
Echocardiographic Findings				
LAD (mm)	39.2 ± 6.4	39.8 ± 6.4	38.1 ± 4.6	0.641
LA volume index (ml/m2)	42.1 ± 13.4	41.5 ± 11.6	38.9 ± 7.1	0.338
IVSTd (mm)	0.89 ± 0.18	0.89 ± 0.16	0.95 ± 0.15	0.545
LVDd (mm)	48.2 ± 4.9	48.1 ± 5.5	46.9 ± 3.9	0.463
EF (%)	60.0 ± 7.3	59.0 ± 6.3	60.0 ± 5.5	0.466
EPS parameters				
AA (mm)	844.8 ± 176.7	867.3 ± 159.9	822.8 ± 148.4	0.465
AH (ms)	95.1 ± 24.0	94.7 ± 26.7	86.5 ± 28.7	0.371
HV (ms)	42.8 ± 24.1	42.0 ± 11.5	39.2 ± 8.4	0.513
AVN ERP (ms)	301.8 ± 73.8	317.9 ± 93.3	304.3 ± 50.3	0.123
A ERP (ms)	229.6 ± 36.7	237.8 ± 51.2	207.5 ± 37.6	0.796
SNRT (ms)	1362.6 ± 405.4	1383.7 ± 312.1	1312.2 ± 287.4	0.901

Binary variables were compared by χ2 test under the allelic model, and quantitative traits were tested for association by linear regression. Data was mean ± SD

*BMI* body mass index, *PAF* paroxysmal atrial fibrillation, *AHI* Apnea Hypopnea Index, *LAD* left atrial diameter, *IVSTd* interventricular septal thickness at end-diastole, *LVDd* LV Dimension diastolic diameter, *EF* ejection fraction, *AVN* atrioventricular node, A atrium, *SNRT* sinus node recovery time

**Fig. 2:**
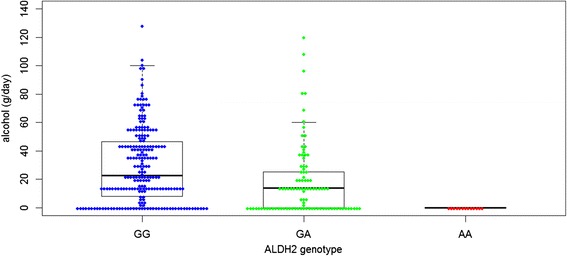
ALDH2 variant (rs671) genotypes and amounts of alcohol intake. A significant linear correlation was found between ALDH2 genotype and mean alcohol intake (*P* = 1.7 × 10^−6^)

Among AF subjects whose alcohol consumption was available, the mean ethanol consumption was 30.6 ± 27.8 g/day in AF patients with GG genotype (*N* = 219), 17.6 ± 24.6 g/day in those with GA genotype (*N* = 98) and 0 g/day in those with AA genotype (*N* = 13). A significant linear correlation was found between *ALDH2* genotype and mean alcohol intake (*P* = 1.7 × 10^−6^) (Fig. 2). In contrast, we found that the frequencies of the *ADH1B* genotypes were similar in patients with AF and in controls.

**Fig. 3:**
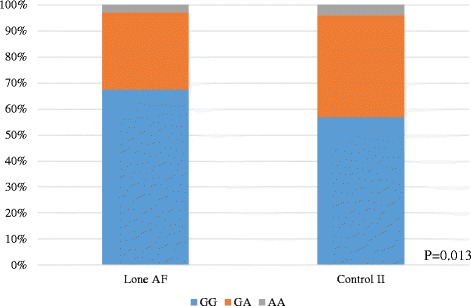
ALDH2 Polymorphism (rs671) in Lone AF Patients and Control II. The dysfunctional genotypes with allele A of ALDH2 SNP rs671 significantly decreased also in Lone AF patients (*P* = 0.01)

**Fig. 4:**
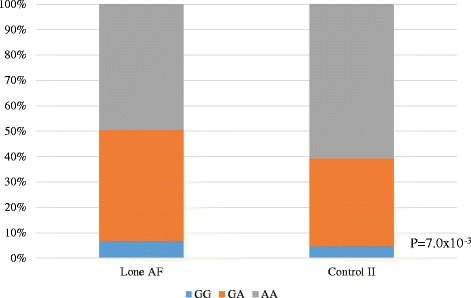
ADH1B Polymorphism (rs1229984) in Lone AF Patients and Control II. The dysfunctional genotypes with allele G of ADH1B SNP rs1229984 significantly increased in lone AF patients (*P* = 7.0x10-4)

A subgroup analysis of 182 patients with lone AF and 914 control subjects (control II), revealed that both *ALDH2* (rs671) and *ADH1B* (rs1229984) were significantly associated with lone AF. The frequency of the dysfunctional allele A of *ALDH2* SNP was significantly lower and the frequency of the dysfunctional allele G of *ADH1B* SNP was significantly higher in patients with lone AF than in control II (*ALDH2* A allele frequency = 0.176 vs 0.235, OR = 1.3, *P* = 0.013, Fig. 3 and Table 3, *ADH1B* SNP G allele frequency = 0.286 vs 0.220, OR = 1.4, *P* = 0.0007, Fig. 4 and Table 4). For the ALDH2 SNP, the GG, GA and AA genotypes were 56.9, 36.2 and 3.9% in control II group as compared to 67.6, 39.2 and 2.7%, respectively, in the lone AF group for *ALDH2*. As for *ADH1B*, the GG, GA and AA genotypes were 4.7, 34.7 and 60.6% in control II group, as compared to 6.6, 44.0 and 49.5%, respectively, in the lone AF group. It is noteworthy that the frequency of the slower alcohol metabolizing allele G in *ADH1B* (rs1229984) was higher in patients with lone AF than in control II subjects. The characteristics of patients with lone AF and *ADH1B* genotypes are shown in Table 5. Among lone AF subjects whose alcohol consumption was available, mean ethanol consumption was 45.1 ± 33.5 g/day in lone AF patients with GG genotype (*N* = 6), 27.8 ± 27.6 g/day in those with GA genotype (*N* = 32) and 22.7 ± 24.3 g/day in those with AA genotype (*N* = 60). Alcohol intake tended to be higher in those with the GG genotype. A significant linear correlation was found between *ADH1B* genotype and the refractory period of the right atrium, indicating atrial conduction disturbance (*P* = 0.0453).

**Table 3:** ALDH2 Polymorphism (rs671) in Lone AF Patients and Control II

	Genotype distribution			HW-test	allelic model (G vs A)			dominant model (GG + GA vs AA)		recessive model (GG vs GA + AA)	
	GG	GA	AA	*P*	A allele frequency	*P*	OR(95%CI)	*P*	OR(95%CI)	*P*	OR(95%CI)
Lone AF	123	54	5	0.74	0.18	1.3x10^−2^	0.7 (0.5–0.9)	0.43	0.7 (0.3–1.8)	0.7x10^−2^	0.6 (0.5–0.9)
	(67.6%)	(29.7%)	(2.7%)								
Control II	520	358	36	0.01	0.24						
	(56.9%)	(39.2%)	(3.9%)								

Control II means control without hypertension or organic heart diseases

The results were tested by the chi-square test and the Cochran–Armitage trend test

The G allele was considered as the reference allele in the allelic model. The A allele was dysfunctional allele

The dysfunctional allele A of ALDH2 SNP rs671 significantly decreased in Lone AF patients (OR 0.7, *P* = 0.01)

*OR* odds ratio

**Table 4:** ADH1B Polymorphism (rs1229984) in Lone AF Patients and Control II

	Genotype distribution			HW-test	allelic model (G vs A)			dominant model (GG + GA vs AA)		recessive model (GG vs GA + AA)	
	GG	GA	AA	*P*	G allele frequency	*P*	OR(95%CI)	*P*	OR(95%CI)	*P*	OR(95%CI)
Lone AF	12	80	90	0.30	0.29	7.0x10^−3^	1.4 (1.1–1.8)	0.005	1.6 (1.1–2.2)	0.28	1.4 (0.7–2.7)
	(6.6%)	(44.0%)	(49.5%)								
Control II	43	317	554	0.78	0.22						
	(4.7%)	(34.7%)	(60.6%)								

The results were tested by the chi-square test and the Cochran–Armitage trend test

The A allele was considered as the reference allele in the allelic model. The G allele was the dysfunctional allele

The dysfunctional allele G of ADH1B SNP rs1229984 significantly increased in lone AF patients (OR 1.4, *P* = 7.0x10-4)

*OR* odds ratio

**Table 5:** Characteristics of Lone AF Patients and ADH1B Genotype

rs1229984 (Dysfunctional Allele = G)	G/G	A/G	A/A	*P*
No. of patients	12	80	90	
Clinical Characteristics				
Age (year)	61 ± 11	61 ± 10	61 ± 11	0.959
Age of onset (year)	58 ± 11	56 ± 12	57 ± 11	0.780
Male gender	10 (83.3%)	66 (82.5%)	79 (87.8%)	0.827
BMI (kg/m2)	23.7 ± 2.7	24.3 ± 3.3	23.9 ± 3.4	0.775
PAF	8 (66.7%)	45 (56.3%)	55 (61.1%)	0.857
Diabetes mellitus	0 (0%)	16 (20.0%)	15 (16.7%)	0.091
Ischemic heart disease	0 (0%)	1 (1.2%)	0 (0%)	0.898
AHI/hour	19.1 ± 11.9	15.7 ± 15.0	18.8 ± 14.5	0.389
Alcohol intake (ethanol g/day)	45.1 ± 33.5	27.8 ± 27.6	22.7 ± 24.3	0.057
Echocardiographic Findings				
LAD(mm)	34.2 ± 10.8	37.1 ± 6.4	37.4 ± 5.5	0.262
LA volume index (ml/m2)	37.8 ± 6.1	37.8 ± 11.7	39.4 ± 9.6	0.346
IVSTd (mm)	0.90 ± 0.12	0.83 ± 0.14	0.89 ± 0.14	0.134
LVDd (mm)	51.7 ± 4.8	46.9 ± 3.9	48.5 ± 5.4	0.525
EF(%)	61.6 ± 5.8	59.3 ± 5.7	58.0 ± 7.6	0.109
EPS parameters				
AA (mm)	861.3 ± 207.3	829.2 ± 152.8	831.3 ± 147.8	0.600
AH (ms)	114.5 ± 9.2	91.7 ± 3.1	93.1 ± 2.3	0.316
HV (ms)	45.6 ± 9.5	39.6 ± 8.5	40.5 ± 8.8	0.693
AVN ERP (ms)	289 ± 64	151 ± 24	155 ± 28	0.117
A ERP (ms)	290.0 ± 40.0	238 ± 38.9	234 ± 29.8	0.0453*
SNRT (ms)	1553.2 ± 622.6	1382.2 ± 448.6	1390.9 ± 326.1	0.643

Binary variables were compared by χ2 test under the allelic model, and quantitative traits were tested for association by linear regression. Data was means, *P < 0.05 by liner regression test

*BMI* body mass index, *PAF* paroxysmal atrial fibrillation, *AHI* Apnea Hypopnea Index, *LAD* left atrial diameter, *IVSTd* interventricular septal thickness at end-diastole, *LVDd* LV Dimension diastolic diameter, *EF* ejection fraction, *AVN* atrioventricular node, A right atrium, *SNRT* sinus node recovery time, *ERP* effective refractory period

## Discussion

*ADH1B* (rs1229984) and *ALDH2* (rs671) are common functional SNPs of alcohol and acetaldehyde-metabolizing enzymes that are prevalent in East Asians [10]. More than 90% of Japanese people, but under 20% of Caucasians, have the A allele of *ADH1B* SNP (rs1229984), which is associated with high metabolic activity in the conversion of alcohol to acetaldehyde. Whereas, about half of Japanese people, but less than 1% of Caucasians, have the dysfunctional A allele of *ALDH2* SNP (rs671), which is associated with deficient metabolic activity in the conversion of acetaldehyde to acetic acid. Few Japanese people have the slower ADH1B G allele [8, 16]. Thus, people with the combination of high *ADH1B* activity and low *ALDH2* activity are very common in Japan.

*ALDH2* is a key enzyme not only for acetaldehyde metabolism, but also for the removal of toxic aldehydes, such as lipid peroxidation-derived 4-HNEand protection against ROS [5, 6]. The dysfunctional A allele of *ALDH2* has been associated with risks for many diseases, including esophageal cancer, coronary artery diseases, Alzheimer’s disease, diabetes, stroke, and others [11, 17].

AF is the most common arrhythmia, and its frequency increases with age. Many risk factors for AF have been reported, including aging, male sex, hypertension, valve disease, left ventricular dysfunction, obesity, sleep apnea, and alcohol consumption [1]. Meta-analyses and prospective studies have established a relationship between alcohol consumption and AF. [2, 3] Several mechanisms for this relationship have been suggested, including hyper adrenergic state [18], impairment of vagal tone [19], a direct effect on myocardial structure [20], and an increase in intra-atrial conduction time [21]. However, no definitive mechanism has been completely elucidated.

We investigated the association of *ADH1B* and *ALDH2* SNPs with AF in Japanese patients undergoing AF ablation. The frequency of the dysfunctional A allele of *ALDH2* was lower in patients with AF than in controls. We also extracted the data from patients with lone AF to eliminate other confounding factors and re-analyzed the data. The frequency of the dysfunctional A allele of *ALDH2* was also lower and the frequency of the slower alcohol metabolizing G alleles of *ADH1B* was higher in patients with AF than in non-AF controls. Contrary to our expectation based on the function of *ALDH2* in reducing oxidative stress, the low frequency of the dysfunctional A allele of *ALDH2* in patients with AF was an unexpected result. This is in contrast with the reports that the *ALDH2* dysfunctional A allele was associated with coronary spastic angina and ischemic stroke in Ease Asian populations [22, 23]. We also noticed that in our study, frequency of ischemic heart diseases was higher in AF patients with AA and GA genotypes than those with the GG genotype-.

Considering that alcohol consumption can cause AF, it stands to reason that patients with the dysfunctional A allele of *ALDH2*, who have a low tolerance for alcohol consumption, were less likely to develop AF. Whereas people with the slower alcohol metabolizing G allele of *ADH1B* were more prone to be heavy drinkers which could lead to a higher risk of AF.

One of the limitations of this study is that quantitative information on alcohol consumption was not fully available for all patients with AF and controls Therefore, we could not determine whether the results were due to *ALDH2* and *ADH1B* SNPs themselves or due to different amounts of alcohol intake. However, in patients with AF, when all the other physiological parameters were similar, we found that there was a significant linear correlation between *ALDH2* genotype and mean alcohol intake. Liu J reported that *ADH1B* and *ALDH2* SNPs were significantly associated with pathogenesis of hepatocellular carcinoma medicated through alcohol drinking [24]. Our limitation was that we were not able to analyze relationship between pathogenesis of AF and these 2 SNPs in stratified with amount of alcohol drinking.

From perspectives that mitochondrial *ALDH2* reduce oxidative stress and the ROS is an important risk factor for AF, low dysfunctional allele of *ALDH2* SNP in AF patients came as an unexpected result. The 4-HNE, accumulated in people with minor allele of *ALDH2* SNP reported to be suppress protein kinase C (PKC) [25]. The mitochondrial ROS causes a reduction of sodium channel and the effect was prevented by inhibition of PKC [26]. The suppression of PKC in people with minor allele of *ALDH2* SNP may work protective for AF occurrence. However, the precise mechanism, of different frequencies of *ALDH2* SNP in AF or non-AF patients have not been elucidated. Future studies will be needed to clarify whether *ALDH2* SNP by itself or alcohol consumption contributes to the occurrence of AF.

In patients with the slow alcohol metabolizing genotype of *ADH1B*, alcohol consumption tended to be higher and the right atrium refractory period was longer than in those with other genotypes. Slow metabolism of blood ethanol by a reduced alcohol elimination rate have been reported in Japanese alcoholic men with the minor G allele of *ADH1B* [27, 28]. Horakova et al. reported that the effects of ethanol on atrial *I*_*k1*_ were heterogeneous, and the heterogeneity of electrical properties might increase susceptibility to AF. [29] The decreased metabolic conversion of alcohol to acetaldehyde and the low elimination rate of alcohol in patients with the slow alcohol metabolizing allele of *ADH1B* may therefore be related to the occurrence of AF in Japanese and other East Asian populations.

There were some limitations in this study. The study was a retrospective, single-center study, and the numbers of cases and controls were small. The main limitation was that information on alcohol consumption was not fully available for both patients with AF and controls. Therefore, we could not clarify whether the relationships between AF and *ALDH2* and *ADH1B* SNPs were caused by SNPs themselves or by different amounts of alcohol intake. It will be necessary to compare the frequencies of *ALDH2* and *ADH1B* SNPs in nondrinking patients with AF and nondrinking controls and stratify the analysis according to alcohol consumption.

Another limitation was that all AF patients included in this study as the AF patients were patients who undergo catheter ablation. They may not necessarily represent all patients with atrial fibrillation. The AF patient undergone AF ablation may be serious patients whose duration of AF was long and resistant for anti-arrhythmic.

The other limitation was that the patients included in this study were all Japanese, we did not consider racial differences.

The interaction of *ALDH2* with *ADH1B* SNPs in alcohol metabolism and multiple risk factors for AF complicated the effects of these SNPs on the occurrence of AF; a precise, large-scale cohort study will be needed to clarify the mechanism.

## Conclusions

*ALDH2* SNP rs671 A allele was negatively associated with AF and *ADH1B* SNP rs1229984 G allele was positively associated with AF. The *ADH1B* SNP rs1229984 may be a marker for susceptibility to AF, and prolonged metabolic conversion of alcohol to acetaldehyde may be related to the occurrence of AF in Japanese and other East Asian populations. A prospective cohort study with a larger sample size for each subgroup of genotypes and alcohol consumption data will be needed to confirm our results.

## Abbreviations

4-HNE: 4-hydroxy-2-nonenal
*ADH1B*: Alcohol dehydrogenase
AF: Atrial fibrillation
AH: Right atria to his
*ALDH2*: Aldehyde dehydrogenase 2
CIs: Confidence intervals
EKG: Electrocardiogram
HV: His to right ventricule
OR: Odds ratio
SNPs: Single nucleotide polymorphisms
